# Modeling how reversal of immune exhaustion elicits cure of chronic hepatitis C after the end of treatment with direct‐acting antiviral agents

**DOI:** 10.1111/imcb.12161

**Published:** 2018-06-05

**Authors:** Subhasish Baral, Rahul Roy, Narendra M Dixit

**Affiliations:** ^1^ Department of Chemical Engineering Indian Institute of Science Bangalore Karnataka India; ^2^ Centre for Biosystems Science and Engineering Indian Institute of Science Bangalore Karnataka India; ^3^ Molecular Biophysics Unit Indian Institute of Science Bangalore Karnataka India

**Keywords:** Bistability, EOT^+^/SVR, mathematical model, sustained virological response, viral dynamics

## Abstract

A fraction of chronic hepatitis C patients treated with direct‐acting antivirals (DAAs) achieved sustained virological responses (SVR), or cure, despite having detectable viremia at the end of treatment (EOT). This observation, termed EOT ^+^/SVR, remains puzzling and precludes rational optimization of treatment durations. One hypothesis to explain EOT ^+^/SVR, the immunologic hypothesis, argues that the viral decline induced by DAAs during treatment reverses the exhaustion of cytotoxic T lymphocytes (CTLs), which then clear the infection after treatment. Whether the hypothesis is consistent with data of viral load changes in patients who experienced EOT ^+^/SVR is unknown. Here, we constructed a mathematical model of viral kinetics incorporating the immunologic hypothesis and compared its predictions with patient data. We found the predictions to be in quantitative agreement with patient data. Using the model, we unraveled an underlying bistability that gives rise to EOT ^+^/SVR and presents a new avenue to optimize treatment durations. Infected cells trigger both activation and exhaustion of CTLs. CTLs in turn kill infected cells. Due to these competing interactions, two stable steady states, chronic infection and viral clearance, emerge, separated by an unstable steady state with intermediate viremia. When treatment during chronic infection drives viremia sufficiently below the unstable state, spontaneous viral clearance results post‐treatment, marking EOT ^+^/SVR. The duration to achieve this desired reduction in viremia defines the minimum treatment duration required for ensuring SVR, which our model can quantify. Estimating parameters defining the CTL response of individuals to HCV infection would enable the application of our model to personalize treatment durations.

## Introduction

The treatment of chronic hepatitis C virus (HCV) infection is undergoing a paradigm shift with the long‐standing combination of pegylated interferon and ribavirin (PR) being replaced by direct‐acting antiviral agents (DAAs).^1^ Whereas PR required 24–48 weeks of therapy and cured about 50% of chronic HCV patients treated, DAA combinations elicit nearly 100% cure with 12 weeks or less of therapy.^2^ Significant efforts are ongoing now to reduce the duration of treatment with DAAs without compromising treatment outcomes.^3^, ^4^ Indeed, treatments as short as 3 weeks have been shown to succeed in select populations.^4^ A promising avenue for such reduction arises from the observation, made by several recent studies, that some patients treated with DAAs had detectable viremia at the end of treatment (EOT) but eventually attained a sustained virological response (SVR), or cure, without additional therapy.^3^, ^5^, ^6^, ^7^, ^8^, ^9^, ^10^, ^11^, ^12^ (Typically, SVR refers to undetectable viremia 12 weeks after the EOT.) For instance, in one study where 240 patients were treated with DAAs for 12 weeks, of the 86 patients available for follow‐up, 22 had detectable viremia at the EOT and 20 of the latter patients eventually achieved SVR.^12^ This observation is in striking contrast to PR treatment, where detectable viremia at the EOT typically implies treatment failure and results in viral rebound.^13^ The achievement of SVR despite detectable viremia at the EOT, a phenomenon termed EOT^+^/SVR, suggests that DAA treatments can be terminated much earlier than the currently prescribed duration of 12 weeks, even if virus is detectable, at least in some patient subpopulations. The benefits of reduced costs and toxicities are expected to be tremendous.^14^ Identifying suitable patient subpopulations and the corresponding reduced treatment durations requires an understanding of the mechanism(s) by which DAA treatments lead to EOT^+^/SVR.

Two hypotheses have been proposed to explain EOT^+^/SVR.^9^ According to the first hypothesis, termed the “virologic” hypothesis, DAAs are argued to render virions produced from infected cells increasingly non‐infectious, to a point where the virions detected at the EOT are incapable of establishing a sustained infection. The residual, predominantly non‐infectious virions would be cleared eventually, leading to SVR. Evidence for this hypothesis comes from studies that show a reduction in viral infectivity following exposure to DAAs, including a preferential targeting of infectious over non‐infectious virion production by some DAAs.^15^, ^16^, ^17^ Furthermore, mathematical models of viral kinetics that account for this mode of DAA action have been constructed and shown to describe data of viral load changes during therapy in patients who experience EOT^+^/SVR, as well as SVR rates elicited by certain DAA combinations.^18^, ^19^ The hypothesis, however, remains to be established conclusively. In patients who achieve EOT^+^/SVR, the virions detectable at the EOT are yet to be shown to be predominantly non‐infectious. The limit of detection with the current assays is 1–10 IU mL^−1^,^3^ which implies the presence of >10^4^–10^5^ virions, given the 15 L of fluid in an average human, at the EOT in patients displaying EOT^+^/SVR. SVR is thought to be achieved when the viral level reaches the “cure boundary” of 1 virion in 15 L.^20^, ^21^ A reduction in viral load of 4–5 log_10_ is thus necessary to achieve SVR following the EOT, which may require several weeks or more given the rate of viral load decline at the EOT.^18^, ^19^ The half‐life of DAAs is 1–2 days,^22^ indicating that the reduction occurs largely in the absence of DAAs. That any new virions produced from infected cells in the absence of DAAs past the EOT are also predominantly non‐infectious remains to be established.

The second hypothesis, termed the “immunologic” hypothesis, argues that treatment with DAAs lowers viral levels, which reverses the exhaustion of effector CD8^+^ T cells (CTLs) induced by chronic exposure to viral antigen, thereby allowing the immune system to clear the residual virus after the EOT. Multiple lines of evidence support this hypothesis: *Ex vivo* studies demonstrate increased exhaustion of CTLs in chronic HCV patients pre‐treatment compared to uninfected individuals and a reduction of this exhaustion accompanied by improved proliferative capacity and functionality of HCV‐specific CTLs during treatment.^23^, ^24^, ^25^ Interferon treatment is unable to induce this reversal,^26^ explaining the absence of EOT^+^/SVR with PR treatment, barring a few exceptions.^27^, ^28^, ^29^, ^30^ When the percentage of hepatocytes infected was reduced below a threshold, exhausted CTLs regained their functionality in a mouse model of HCV infection.^31^ Anti‐PD‐1 therapy, which prevents or reverses CTL exhaustion, has shown some success in HCV‐infected primates and humans.^32^, ^33^ Unlike the virologic hypothesis, however, whether the immunologic hypothesis is consistent with patient data of viral load changes during DAA treatments is unknown. No model of viral kinetics based on the immunologic hypothesis has yet been constructed.

In this study, we constructed a model of viral kinetics based on the immunologic hypothesis, examined its ability to describe published patient data, and assessed its implications for shortening treatment durations. Models of viral kinetics have been used widely to describe viral load changes in patients during treatment, have provided key insights into disease progression and guided treatments.^21^ Existing models, including the recent extensions based on the virologic hypothesis,^18^, ^19^ have focused primarily on describing the impact of drugs. The influence of the immune system in controlling HCV infection is rarely considered explicitly. Models of CTL dynamics and exhaustion have been constructed previously in the context of other chronic infections.^34^, ^35^, ^36^, ^37^ For instance, CTL exhaustion in chronic HIV‐1 infection and its reversal during antiretroviral treatment that leads to lasting post‐treatment control but not clearance of infection in a subset of infected individuals has recently been described using a mathematical model.^36^ Here, we integrated a formalism of CTL exhaustion, drawn from the latter models, with a basic model of HCV kinetics to elucidate the immunologic hypothesis underlying EOT^+^/SVR. We found that our model recapitulates data of viral load changes in patients experiencing EOT^+^/SVR, identifies an underlying bistability in the system as the origin of EOT^+^/SVR, and presents an avenue, exploiting the bistability, to estimate the minimum DAA treatment durations required to achieve SVR.

## Results

### Mathematical model

**Figure 1:**
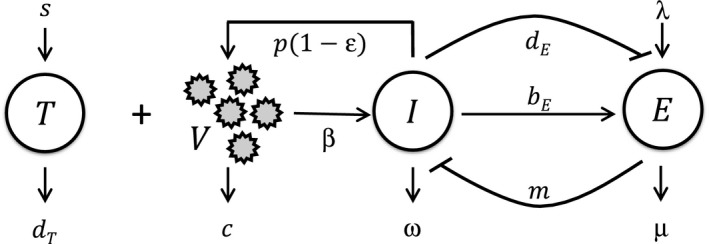
Schematic of the model. Target cells, *T*, are infected by free virions, *V*, to yield infected cells, *I*, which produce new virions. Treatment with DAAs lowers this production of new virions. *I* activate effector cells, *E*, which in turn control *I*. *I*, however, can also suppress *E* by inducing their exhaustion. The model parameters and equations governing the ensuing dynamics are described in the text.

We constructed a model of HCV kinetics in a chronically infected individual under DAA therapy with CTL dynamics governed by the immunologic hypothesis (Figure 1). The following equations described the ensuing dynamics and predicted viral load changes in the individual during and after therapy:

(1) $$\frac{dT}{dt}=s-d_{T}T-\beta VT$$

(2) $$\frac{dI}{dt}=\beta VT-\omega I-mEI$$

(3) $$\frac{dV}{dt}=p\left(1-\varepsilon \left(t\right)\right)I-cV$$

(4) $$\frac{dE}{dt}=\lambda +b_{E}\frac{I}{k_{B}+I}E-d_{E}\frac{I^{n}}{k_{D}^{n}+I^{n}}E-\mu E$$

Here, following the basic model of HCV kinetics,^21^ we let target cells, *T*, be produced at the rate *s* and lost with a first order rate constant *d*_*T*_. Free virions, *V*, infect target cells with the second order rate constant *β*, yielding infected cells, *I*. In the absence of treatment, infected cells produce virions at the rate *p* per cell. DAAs lower *p* by the factor 1‐ε(*t*), where ε(*t*) is the efficacy of treatment at time *t* following the start of treatment. For simplicity, we assumed that the efficacy is constant during treatment and rapidly vanishes after the EOT, so that ε(0 ≤ *t ≤ τ*_*d*_) = ε and ε(*t>τ*_*d*_) = 0, where *τ*_*d*_ is the treatment duration. Virions are cleared with the rate constant *c*. Infected cells are killed by HCV‐specific CTLs, *E*, with the second order rate constant *m*. The loss of infected cells by mechanisms independent of *E* is assumed to occur with the first order rate constant ω. To describe CTL dynamics, we followed previous studies,^34^, ^36^ where CTLs are assumed to be produced at the rate λ and are lost with the first order rate constant μ. They are activated by contact with infected cells with the second order rate constant *b*_*E*_. This activation is saturable and is half‐maximal when *I* equals *k*_*B*_. Sustained stimulation or stimulation with high levels of antigen induces CTL exhaustion, which we described using a Hill function with the second order rate constant *d*_*E*_, the exponent *n*, and the half‐maximal value *k*_*D*_. As *I* increases from values much smaller to much larger than *k*_*D*_, the level of exhaustion rises from minimal to maximal. The larger the value of *n*, the sharper this rise is around *k*_*D*_.

The equations above present a model of HCV kinetics that incorporates the essential features of the immunologic hypothesis underlying EOT^+^/SVR. Incorporating additional features such as target and infected cell proliferation or cumulative antigenic stimulation leading to exhaustion did not alter our results (Supplementary text 1, Supplementary text 2, Supplementary figure 1, and Supplementary figure 2).

### The immunologic hypothesis and EOT^+^/SVR

**Figure 2:**
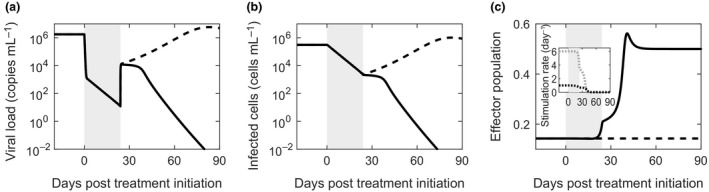
Model prediction of EOT ^+^/SVR. Changes in **(a)** the viral load, **(b)** the infected cell population and **(c)** the effector population in a chronically infected individual as a function of time from the start of treatment with DAAs predicted by our model with (solid) and without (dashed) the immunologic hypothesis. The rates of activation (black dotted) and exhaustion (gray dotted) of effector cells, defined by the second and third terms on the right‐hand side of Equation (4), respectively, corresponding to the case with the immunologic hypothesis are shown in the inset in **c**. The treatment duration is shaded. The parameter values employed are in Table 1 and correspond to those in Figure 3b.

To examine whether the immunologic hypothesis yielded EOT^+^/SVR, we solved the above model equations with parameter values representative of chronic HCV patients (Table 1; Methods) and predicted viral load changes during and after therapy. We found that the model yielded EOT^+^/SVR (Figure 2). The viral load declined in a biphasic manner during therapy (Figure 2a), as observed in patients.^10^, ^11^ From the classical analysis of the basic model of viral kinetics,^21^, ^38^, ^39^ the first phase is attributed to the decreased production of virions from infected cells due to drug action, which destroys the pre‐treatment balance between viral production and clearance (*pI*_0_ = *cV*_0_) in the chronic phase of infection. The second phase is attributed to the resulting loss of infected cells (Figure 2b). Viral production and clearance are estimated to be fast relative to infected cell production and loss, leading to a pseudo steady state between virion and infected cell populations in the second phase [*p*(1‐*ε*)*I* ≈ *cV*].

**Table 1:** Parameters and their values

Parameter	Meaning	Value [95% CI]	Source
*s*	Target cell production rate	10^5^ cells mL^−1^ day^−1^	14, 18
*d*_*T*_	Target cell death rate	0.01 day^−1^	39
β	Infectivity	1 × 10^−8^ mL cells^−1^ day^−1^	14, 18, 51
ω	Natural death rate of infected cells	0.01 day^−1^	ω = *d*_*T*_
λ	Effector cell recruitment rate	1 cells mL^−1^ day^−1^	36
*b*_*E*_	Antigen‐induced proliferation rate of effector cells	1 day^−1^	
μ	Death rate of effector cells	2 day^−1^	
*k*_*B*_	Hill function scaling for effector cell proliferation	10^3^ cells mL^−1^	35, 36
*n*	Hill coefficient for exhaustion	>1	
*p*	Production rate of virions by infected cells	4.4 virions cell^−1^ day^−1^	$p=\frac{d_{T}\delta c}{s\beta }\left(\frac{V_{0}\beta }{d_{T}}+1\right)$
*m*	Killing rate of infected cells by effector cells	0.244 mL cells^−1^ day^−1^	$m=\frac{\delta -\omega }{E_{0}}$
δ	Death rate of infected cells	0.059 [0.026–0.092] day^−1^	Best‐fits (Figure 3a)^a^
ε	Efficacy of drugs	0.9991 [0.9988–0.9994]	
*c*	Viral clearance rate	6.3 [5.8–6.8] day^−1^	
*d*_*E*_	Antigen‐induced exhaustion rate of effector cells	4 day^−1^	
*k*_*D*_	Hill function scaling for effector cell exhaustion	2.7 × 10^4 ^cells mL^−1^	

a For the patient in Figure 3b, δ = 0.2 day^−1^, *d*_*E*_ = 6 day^−1^, *k*_*D*_ = 1.95 × 10^3^ cells mL^−1^, *p *=* *35.7 virions cell^−1^ day^−1^, and *m *=* *1.36 mL cells^−1^ day^−1^.

Following the EOT, drug action ceased and viral production from infected cells was restored to its pre‐treatment rate. The pseudo steady balance between viral production and clearance was again lost, but now in the opposite sense, leading to a surge in viremia. An “inverse” of the first phase decline thus resulted. A new pseudo steady state of virion and infected cell populations was established at a much higher viremia corresponding to the increased viral production rate from infected cells (*pI* ≈ *cV*).

The subsequent dynamics was determined by the CTL response. During treatment, in accordance with the immunologic hypothesis, the reduction in viremia resulted in an increase in the CTL population (Figure 2c). Note that in our model, as in previous formalisms,^35^, ^36^ the variable *E* is a scaled variable, allowing a compact representation of the net effect of CTLs subsuming the CTL population size and the level of CTL exhaustion. For simplicity, we refer to *E* as the CTL population. The product *mE* represents the rate of killing of infected cells by CTLs, where *m* links *E* to absolute rates of infected cell death. As we show below, the value of *mE* we estimate is consistent with independent estimates of CTL killing of infected hepatocytes. The increase in the CTL population above was due to a reduction in the rate of CTL exhaustion (Figure 2c, inset). The increased CTL population during treatment turned out to be sufficient not to let the increase in viremia past the EOT be long‐lasting. CTL action steadily controlled viremia past the EOT (Figure 2a), further reducing the level of exhaustion (Figure 2c, inset). Eventually, the competition between CTL activation and exhaustion by infected cells was resolved in favor of activation, resulting in a significant rise in the CTL pool (Figure 2c). This larger CTL pool destroyed the infected cell population (Figure 2b), causing a sharp decline in viremia and eventually leading to SVR (Figure 2a). As the infection was cleared, the CTL population declined and reached a new steady state corresponding to the level in the absence of infection and exhaustion (Figure 2c). (Note that immunological memory is not explicitly considered in this model, in keeping with extant models of CTL exhaustion.^34^, ^36^)

To confirm the influence of the immunologic hypothesis in our predictions of EOT^+^/SVR, we solved the model equations also in the absence of the immunologic hypothesis. Here, the CTL population was held fixed at the pre‐treatment level (*E = E*_0_) and no reversal of exhaustion was allowed. The model effectively reduced to the basic model of viral kinetics.^21^ The viral level rose after the EOT and eventually reached pre‐treatment levels, marking the failure of treatment (Figure 2a–c, dashed lines).

### Comparisons of model predictions with patient data

We next compared model predictions to data of viral load changes in two patients who achieved EOT^+^/SVR following DAA‐based therapy of ultra‐short durations^10^, ^11^ (see Methods). We assumed that the restoration of exhausted CTLs was negligible in the short period during therapy (~3 weeks) and fit the resulting reduced, basic model of viral kinetics to the data during therapy. The best‐fit predictions described patient data well (Figure 3). (Sensitivity analysis indicated that parameter estimation could be done robustly; see Supplementary figure 3). The best‐fit parameter values (Table 1) are consistent with previous estimates. For instance, the viral clearance rate, *c *=* *6.3 day^−1^, is close to the reported^38^ average of 6.2 ± 1.8 day^−1^; the drug efficacy, ε = 0.9991, is consistent with the mean of 0.997 estimated for sofosbuvir‐based treatments^14^, ^18^; and the death rate of infected cells, δ = 0.06 day^−1^ and 0.2 day^−1^ for the two patients, is in the range of values, 0.01–0.4 day^−1^, reported earlier.^20^

**Figure 3:**
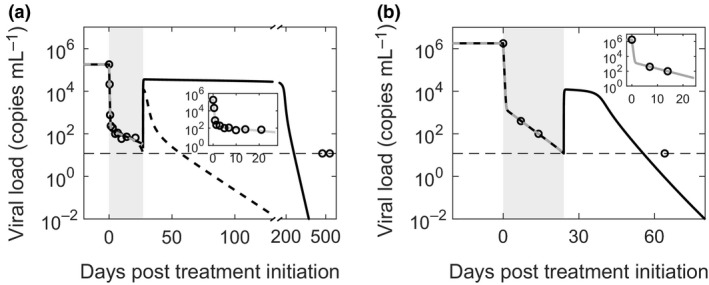
Comparisons with patient data. Model predictions (solid lines) compared with data (symbols) from two patients, shown separately in **a** and **b**, who achieved EOT ^+^/SVR (see text for details of the patients). The best‐fits to the data during therapy are shown superimposed (gray dashed lines) and in expanded view (insets). The best‐fit parameter estimates and their 95% CIs are as follows: **(a)** δ = 0.059 (0.026–0.091) day^−1^, ε = 0.9991 (0.9988–0.9994), and *c *=* *6.3 (5.8–6.8) day^−1^. These yielded *p *=* *4.4 virions cell^−1^ day^−1^ and *m *=* *0.24 mL cells^−1^ day^−1^. The minimal values of *d*_*E*_ **= 4 per day and *k*_*D*_ = 2.67 × 10^4^ cells mL ^−1^ (see Methods) yielded SVR just before the last measured time point (solid line). The low second phase slope (δ = 0.059 day^−1^), implying weak effector killing of infected cells, and the long gap between the EOT and the last measurement yielded best‐fit parameters that predicted high viremia over extended durations past the EOT, which may be unrealistic. A low second phase slope may also arise due to infected cell proliferation despite strong effector killing. A model that included cell proliferation (Supplementary text 1; Supplementary figure 1) captured the data well and resulted in rapid viral load reduction past the EOT leading to SVR within a few weeks (dashed line). During the period under treatment, predictions of the two models are indistinguishable. **(b)** δ = 0.204 (0.200–0.208) day^−1^. ε = 0.9991 and *c *=* *6.3 day^−1^ were the same as in **a**. These yielded *p *=* *35.7 virions cell^−1^ day^−1^ and *m *=* *1.36 mL cells^−1^ day^−1^. Furthermore, *d*_*E*_ = 6 day^−1^ and *k*_*D*_ = 1.95 × 10^3^ cells mL ^−1^ were the minimal estimates. The limit of detection is shown as a thin dashed line. The other parameter values employed are in Table 1.

We then estimated parameters defining CTL exhaustion as those that yielded EOT^+^/SVR for the treatment duration employed but not any shorter, ensuring that viremia became undetectable in the predictions within the timeframe reported clinically. With these limiting estimates of the parameters, our model predictions were consistent with the patient data (Figure 3). The predictions during therapy were indistinguishable from those of the basic model, justifying our assumption above. Furthermore, from the estimates of *m* (Table 1) and the evolution of *E*, the rate of loss of infected cells due to CTL killing, *mE*, we estimated was in the range 0.04–0.16 day^−1^ and 0.2–0.8 day^−1^ for the two patients, respectively, which was similar to the rate, 0.14 ± 0.21 day^−1^, estimated independently from an analysis of acute infection in chimpanzees.^40^ Model predictions thus described viral load changes during treatment and the observed EOT^+^/SVR quantitatively. The immunologic hypothesis was thus consistent with patient data.

### Bistability and the origin of EOT^+^/SVR

**Figure 4:**
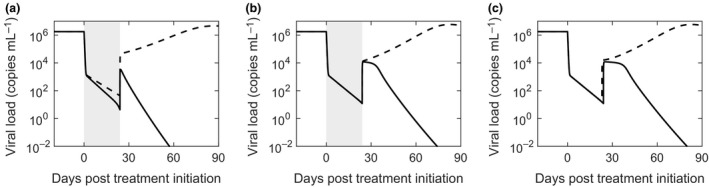
Dependence of EOT ^+^/SVR on parameter values. Model predictions of viral load changes under conditions leading to EOT ^+^/SVR (solid lines) and treatment failure (dashed lines). **(a)** *m *=* *1.5 mL cells^−1 ^day^−1^ (solid) and *m *=* *1 mL cells^−1^ day^−1^ (dashed); **(b)** *k*_*D*_ = 2 × 10^3^ cells mL^−1^ (solid) and 1 × 10^3^ cells mL^−1^ (dashed); **(c)** *m *=* *1.36 mL cells^−1^ day^−1^; *τ*_d_ = 24 days (solid) and 23 days (dashed). The treatment period is shaded in **a** and **b**. The other parameter values employed are in Table 1 corresponding to Figure 3b.

The EOT^+^/SVR phenomenon was sensitive to model parameters. For instance, reducing *m*, the strength of the CTL response, while keeping all other parameters fixed, resulted in viral rebound and treatment failure (Figure 4a). Similarly, reducing *k*_*D*_ resulted in viral rebound (Figure 4b). For parameter values that led to viral rebound, increasing the treatment duration restored EOT^+^/SVR (Figure 4c). The divergent outcomes, viral rebound or SVR, realized with subtle variations in model parameter values or treatment duration implied the existence of bistability in the system.

**Figure 5:**
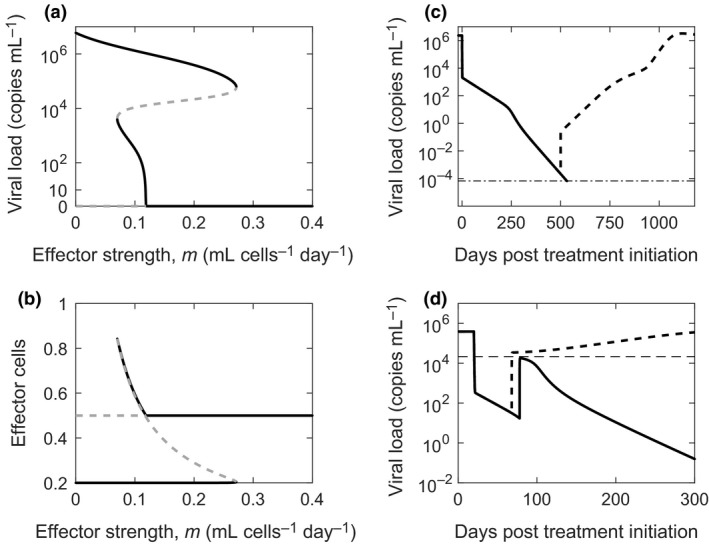
Bistability and treatment outcomes. Bifurcation diagrams indicating stable (black) and unstable (gray) steady state viral loads **(a)** and effector cell populations **(b)** as functions of the strength of the effector response, *m*. Viral load changes during and after therapy when *m *=* *0.055 mL cells^−1^ day^−1^ **(c)** and *m *=* *0.2 mL cells^−1 ^day^−1^ **(d)**. In **c**,* τ*_d_ = 500 days (dashed line) and 552 days (solid line). The thin dot‐dashed black line marks the cure boundary. In **d**,* τ*_d_ = 48 days (dashed line) and 58 days (solid line). The thin dashed line marks the unstable steady state viral load when *m *=* *0.2.

To elucidate the bistability, we solved our model equations at steady state in the absence of treatment (ε = 0). We explored the resulting states as functions of *m*, with all the other parameters constant. We found that when *m* was low, less than ~0.07 mL cells^−1^ day^−1^, a single stable steady state with high viremia, ~10^6^ copies mL^−1^, marking chronic infection, existed (Figure 5a). The corresponding effector population was low, marking significant exhaustion (Figure 5b). (Linear stability analysis is presented in Supplementary text 3 and Supplementary figure 4.) When *m* was between ~0.07 and ~0.27 mL cells^−1^ day^−1^, the system exhibited two stable steady states, chronic infection with high viremia and viral clearance. The effector population was low in the first and high in the second. The stable states were separated by an unstable steady state with intermediate viremia of ~10^4^ copies mL^−1^ and an intermediate effector population. For very high values of *m*, greater than ~0.27 mL cells^−1^ day^−1^, the system was again monostable with viral clearance the only admissible steady state. The corresponding effector population reflected the steady state CTL level in the absence of infection.

We note that over a narrow range of values *m*, ~0.07 to ~0.12 mL cells^−1^ day^−1^, a low viremic stable state arose instead of complete viral clearance. Whether this is akin to the low viremia seen in some patients following SVR with PR treatment^41^ or a consequence of not incorporating other arms of the immune response, such as NK cells, in our model, remains to be established. Parameter values can be identified that preclude the low viremic state in our model (Supplementary figure 5). Note that viremia became undetectable after ~8 years in all the patients with low viremia after SVR barring one who had a reinfection with a different strain of HCV.^41^ Here, we therefore assumed that the low viremic state also represented viral clearance.

Treatment could drive the system from the chronic infection state to clearance. When *m* was small and the system monostable, treatment had to last till the virus was completely cleared, i.e., viremia crossed the cure boundary. Any residual viremia at the EOT would lead to viral rebound and treatment failure. For instance, with *m *=* *0.055 mL cells^−1^ day^−1^, ending treatment even after the viremia was reduced to ~10^−4^ copies mL^−1^ led to viral rebound and treatment failure (Figure 5c). Here, EOT^+^/SVR was not possible. In contrast, when the system was bistable, treatment had to last long enough to drive viremia past the intermediate unstable state to a point where upon cessation of therapy the surge in the viremia due to the inverse first phase did not allow the viremia to cross the unstable boundary again. The system would then spontaneously clear the virus without the need for any treatment. This behavior marked EOT^+^/SVR. For instance, with *m *=* *0.2 mL cells^−1^day^−1^, treatment of 48 days led to the inverse first phase allowing viremia to increase above the unstable boundary of 2.15 × 10^4^ copies mL^−1^, leading to viral rebound and treatment failure, whereas treatment of 58 days kept viremia below the unstable boundary and led to EOT^+^/SVR (Figure 5d). The observed EOT^+^/SVR thus originated from the bistability intrinsic to the system. At the highest values of *m*, chronic infection was not admissible, potentially representing individuals who mount strong CTL responses and spontaneously clear HCV.^42^

### The minimum required treatment duration

**Figure 6:**
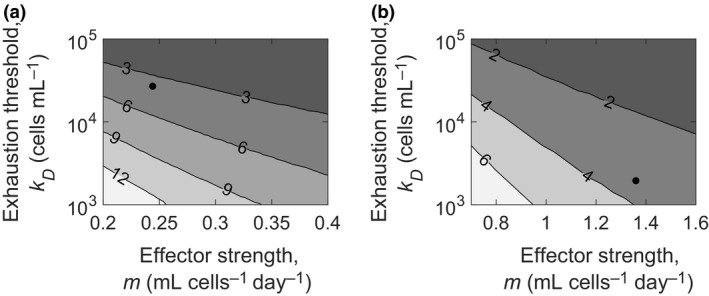
Minimum required treatment duration. Contour plots of model predictions of the minimum treatment duration required in weeks to achieve SVR as functions of the parameters *m* and *k*_*D*_ with the other parameters representative of the patient data in **(a)** Figure 3a and **(b)** Figure 3b. The parameter values defining the patients are shown as black dots.

The calculations above indicated that the minimum treatment duration to achieve EOT^+^/SVR was that required to drive viremia past the intermediate steady state and contain it below the boundary soon after treatment cessation. We solved our model equations to estimate this requisite treatment duration as a function of *m* and *k*_*D*_. Note that although we varied *m* above to illustrate bistability, variations in other model parameters could also yield bistability. Here, we considered *k*_*D*_ additionally, as it was one of the parameters that modulated the level of CTL exhaustion. We varied *m* and *k*_*D*_ over ranges that encompassed values that described the patient data in Figure 3. All the other parameters, including the treatment efficacy, were kept at the values corresponding to the patients. Recall that the patients achieved SVR following 27 days and 24 days of therapy, respectively. With the first patient (Figure 3a), as *m* was lowered, the required treatment duration increased (Figure 6a). Higher values of *m*, marking higher CTL killing rates, required shorter durations. Similarly, decreasing *k*_*D*_, which worsened CTL exhaustion, required longer treatment durations, and vice versa (Figure 6a). We repeated the calculations with parameter values corresponding to the second patient above (Figure 3b), and found the same qualitative behavior (Figure 6b). The treatment durations were different, however, for the two cases, even with the same values *m* and *k*_*D*_, reflecting differences in the other parameters between the two patients.

Thus, given a set of parameter values representing an individual, our model can be applied to estimate the minimum duration of treatment required to achieve EOT^+^/SVR.

## Discussion

An understanding of the intriguing EOT^+^/SVR phenomenon holds promise not only of unraveling new insights into our immune response to HCV, and to pathogens in general, but also of defining new limits on the required durations of DAA treatments. In this study, we constructed a mathematical model of viral kinetics based on the proposed immunologic hypothesis underlying EOT^+^/SVR and found that it recapitulated the EOT^+^/SVR phenomenon and data of viral load changes in patients treated with DAAs who experienced EOT^+^/SVR. Importantly, the model predicted that EOT^+^/SVR originated from the bistability intrinsic to the dynamical system governing the interactions between the virus, target cells, infected cells and effector cells in an infected individual. Furthermore, it showed how, exploiting this bistability, the minimum treatment duration required to realize EOT^+^/SVR can be estimated, presenting an avenue for systematically reducing DAA treatment durations from the current guidelines.

The bistability of the system arose at intermediate strengths of the effector cell response to the infection. When the response was very strong, chronic infection was not established, with the sole steady state marking the clearance of infection. This behavior may be reminiscent of those individuals who clear the infection spontaneously. Strong CTL responses have been implicated in spontaneous clearance,^43^ which happens in an average of ~26% of infected individuals.^42^ When the response was very weak, chronic infection with high viremia was the only steady state possible. Individuals with this behavior may represent those who experience viral rebound after the EOT, whether viremia was detectable at the EOT or not. Cure is possible in such individuals when treatment lasts until viremia drops below the cure boundary. With intermediate strengths of the effector response, both chronic infection and viral clearance became stable steady states separated by an unstable steady state with intermediate viral load. EOT^+^/SVR would result in such patients when treatment drives viremia from the chronic state to below the unstable intermediate state to a point that does not allow resurgence past the unstable boundary following the EOT. The system then spontaneously reaches the stable state of viral clearance. The time it takes for treatment of a given efficacy to achieve the desired reduction in viremia defines the minimum duration for which the treatment must be administered.

The extent of the rise in viremia soon after the EOT can be estimated. The pseudo steady states of viral production and clearance just before and after the EOT imply that the fold‐increase in viremia soon after the EOT would be 1/(1‐ε), where the treatment efficacy ε is readily estimated from early viral kinetics following the start of therapy. The rise in viremia post‐treatment by the factor 1/(1‐ε) has been recognized earlier using the basic model of viral kinetics in the context of PR treatment assuming a constant target cell population.^44^ Whether the resulting viremia would lie above or below the intermediate unstable boundary is more difficult to predict because the boundary is less well estimated. The boundary depends on parameters defining CTL activation and exhaustion dynamics as well as the strength of the CTL response, which remain difficult to measure or estimate. In our model, these would be the parameters *m* in Equation (2) and *b*_*E*_, *k*_*B*_, *d*_*E*_, *k*_*D*_ and *n* in Equation (4). Measurements that allow estimation of these latter parameters would enable the application of our model to specify the unstable boundary and hence define personalized treatment durations. A confounding factor is that measurements of HCV‐specific CTL levels in the blood may not be representative of the effector response in the liver.^45^ CTL‐induced infected cell death rates have been estimated previously by analyzing measurements of serum alanine aminotransferase levels during acute infection in chimpanzees.^40^ Future studies may reveal whether such measurements during the treatment of chronic infection in humans bear signatures of the reversal of exhaustion and hence can be analyzed to estimate the unknown parameters above. The fraction of patients treated who achieve EOT^+^/SVR appears to vary widely across treatments and patient populations.^3^, ^5^, ^6^, ^7^, ^8^, ^9^, ^10^, ^11^, ^12^ Weak effector responses and/or inadequate treatment durations may underlie the inability of patients to achieve EOT^+^/SVR compared to those who do in the latter reports. Again, future studies that may quantify the inter‐patient variations in CTL dynamics and response may help explain the wide variations in the fraction of patients achieving EOT^+^/SVR in the reports above.

By showing that the immunologic hypothesis is consistent with patient data of viral load changes, our study adds an important missing piece to the evidence gathered so far in support of the hypothesis.^23^, ^24^, ^25^, ^31^, ^32^, ^33^ Our study, however, does not negate the alternative, virologic hypothesis. Indeed, the two hypotheses are not mutually exclusive. To evaluate the consistency of the immunologic hypothesis with patient data, we constructed a model that considered the latter hypothesis alone and showed that it was able to capture the same patient data that was captured by a model based on the virologic hypothesis.^18^ While a model can be constructed readily that incorporates both the hypotheses, patient data in addition to viral load changes during therapy, such as viral infectivity or the strength of the effector cell response, may be required to distinguish between the hypotheses. The relative extents to which the two hypotheses contribute to EOT^+^/SVR remains unknown. Delineating the contributions of the two hypotheses in patients undergoing different DAA‐based treatments would lead to a more comprehensive description of EOT^+^/SVR as well as more accurate estimates of the minimum required treatment duration for specific DAA combinations.

EOT^+^/SVR has been achieved recently in a patient treated with DAAs during the acute phase of infection,^46^ where CTL exhaustion may not have set in to the same extent as in the chronic phase of infection. Whether this adds to the evidence in support of the virologic hypothesis or whether sufficient CTL exhaustion had set in by the start of treatment, which was 9 weeks after infection, for the immunologic hypothesis to hold in the patient remains unknown. Similarly, EOT^+^/SVR has also been reported in some individuals treated with PR.^27^, ^28^, ^29^, ^30^ Interferon is argued to have a pleiotropic influence on CTLs, enhancing their proliferation and activation in the early stages of infection and rendering them more exhausted during the late stages of infection.^47^ Indeed, early initiation of PR treatment has been suggested to improve treatment outcomes.^48^ Furthermore, blockade of interferon signaling during chronic LCMV infection of mice has been shown recently to clear the infection.^49^, ^50^ Whether a critical time following infection exists within which the start of PR therapy does not exacerbate CTL exhaustion remains to be ascertained. Low level viremia has been shown to persist for several years in patients successfully treated with PR, arguing further for a role of the immune system in effecting a complete cure of HCV infection.^41^

Our model employed several simplifying assumptions. First, following the basic model of viral kinetics,^21^ DAA efficacy was assumed to be constant during therapy. Pharmacokinetic effects and the accumulation of resistance‐associated mutations,^51^ which would result in a time‐varying drug efficacy, were not considered. Furthermore, DAAs may have multiple modes of antiviral action,^52^ which we ignored. Second, we considered the simplest model of CTL dynamics that includes antigen‐driven activation and exhaustion. The description has been employed successfully to explain the post‐treatment control of HIV‐1 infection.^36^ More sophisticated models that incorporate the dependence of exhaustion on sustained antigenic stimulation have been proposed,^35^, ^37^ but have been argued to have similar qualitative features as the simpler model employed here.^36^ Third, studies have argued that in addition to CTL function, NK cell function is restored during IFN‐free DAA therapy,^53^ which we did not consider. Finally, we did not consider viral evolution leading to immune escape, which may underlie cases such as with the one patient who showed detectable viremia at the EOT, achieved undetectable viremia at week 4 past the EOT, but relapsed at week 12 past the EOT.^9^ Our aim was to construct a model that captured the essential features leading to EOT^+^/SVR according to the immunologic hypothesis and compare its predictions with patient data, which the above simplifications did not compromise.

In summary, our study elucidates the origin of EOT^+^/SVR based on the immunologic hypothesis, describes patient data of viral load changes, and presents an avenue to define minimum DAA treatment durations. Quantifying the extent to which the immunologic hypothesis contributes to EOT^+^/SVR with different DAA combinations, a promising avenue for future studies, would facilitate the identification of optimal durations of DAA‐based treatments.

## Methods

### Parameter estimates and solution of model equations

We employed the following parameter estimates (Table 1). Following previous studies, we set the target cell production and loss rates to *s *= 10^5^ cells mL^−1^ day^−1^ and *d*_*T*_ **= 0.01 day^−1^, corresponding to a target cell population in an uninfected individual of 10^7^ cells mL^−1^,^14^, ^18^, ^39^ and the infection rate of target cells, β = 10^−8^ mL cell^−1^ day^−1^.^14^, ^18^, ^51^ We let the death rate of infected cells other than by CTL killing be the same as the natural death rate of target cells, neglecting any HCV‐induced cytopathicity; thus, ω = *d*_*T*_. We let the CTL production, death and activation rates be λ = 1 cell mL^−1^day^−1^, μ = 2 day^−1^, *b*_*E*_ = 1 day^−1^ and *k*_B_ = 10^3^ cells mL^−1^.^35^, ^36^ The viral production and clearance rates, *p* and *c*, the drug efficacy, ε, the strength of CTL killing, *m*, and the parameters governing CTL exhaustion, *d*_*E*_, *k*_*D*_ and *n*, are expected to vary across individuals. We estimated them by comparisons of model predictions with patient data (see below). We ensured that *k*_*D*_ *> k*_*B*_ to let CTL exhaustion occur at higher antigen levels than CTL activation. We solved model equations in MATLAB with initial conditions marking the pre‐treatment steady state, obtained by setting the left‐hand sides of all the equations to zero. We examined the sensitivity of our model predictions to parameter variations by computing partial rank correlation coefficients^54^ (Supplementary figure 3).

### Patient data

We considered published data of viral load changes in two patients who experienced EOT^+^/SVR with ultra‐short DAA‐based treatments.^10^, ^11^ The same data have been employed in a recent modeling study based on the virologic hypothesis.^18^ One patient received treatment with sofosbuvir and ribavirin for 27 days.^10^ The last reported viral load measurement during therapy was at day 21 from the start of therapy, when viremia was detectable. The patient was then lost to follow up until day 475, when viremia was reported undetectable. The second patient was treated with a combination of peritaprevir, ombitasvir, dasabuvir plus ritonavir and ribavirin for 24 days.^11^ The last reported viral load measurement during therapy was at day 14 from the start of therapy, when viremia was detectable. At the first measurement past the EOT, at day 64 from the start of therapy, viremia was undetectable and remained so for over 7 months past the EOT. Data was digitized using the Engauge digitizer.

### Comparisons of model predictions with patient data

The number of unknown parameters was too large to fit our model directly to the data. We therefore employed the following procedure. The treatments of the two patients were ultra‐short, lasting < 4 weeks. We assumed that during this period, the effector population, *E*, remained approximately equal to its pre‐treatment value, *E*_0_. Equation (4) above could then be ignored, reducing the model to the basic model of HCV kinetics. Fitting the reduced model to patient data from one patient^10^ during treatment then allowed estimation of ε, *c*, and the composite parameter δ = ω + *mE*_0_. With the other patient,^11^ because of infrequent sampling, we set ε and *c* to the values obtained above, which were similar to the values estimated elsewhere,^18^ and fit the data using δ as an adjustable parameter. We performed data fitting using the function NLINFIT in MATLAB.

We estimated the remaining parameters as follows. The pre‐treatment steady state implied that *T*_0_ = *s*/(*d*_*T*_+*βV*_0_), *I*_0_ = *βV*_0_ *T*_0_/δ, *p *= *cV*_0_/*I*_0_ and $E_{0}=\lambda /\left(\mu +d_{E}\frac{I_{0}^{n}}{k_{D}^{n}+I_{0}^{n}}-b_{E}\frac{I_{0}}{k_{B}+I_{0}}\right)$ from Equations (1), (2), (3), (4). Knowledge of the pre‐treatment viral load, *V*_0_, thus allowed estimation of *T*_0_, *I*_0_ and *p* from the first three relationships above. The fourth relationship allowed defining *E*_0_ as a function of the unknown exhaustion parameters, *d*_*E*_, *k*_*D*_ and *n*. Note that because δ = ω + *mE*_0_, this implied that *m* was also defined as a function of the latter three parameters. Many combinations of these latter parameters were likely to yield admissible solutions of our equations. Data of viral load changes past the EOT were not available to define these parameters uniquely. To restrict choices, we let *n *=* *3, a value used previously.^36^ (Using *n *=* *1, also used previously,^36^ did not alter the stability properties; see Supplementary figure 5.) We then identified minimal estimates of *d*_*E*_ and *k*_*D*_ such that EOT^+^/SVR was achieved with the administered treatment duration and no shorter and the viremia became undetectable within the timeframe reported. For this, we first fixed *d*_*E*_ **= 2 day^−1^, also employed previously,^36^ and solved model equations (Equations (1), (2), (3), (4)) for different values of *k*_*D*_ and identified the smallest value of *k*_*D*_ for which the administered treatment duration yielded SVR within 3000 days. We picked 3000 days as an upper bound for SVR based on previous studies where low level viremia was detected with sensitive assays up to 8 years after having received successful PR treatment.^41^ If this was not possible for any value of *k*_*D*_, we increased *d*_*E*_ and repeated the above procedure until the latter criterion was satisfied. We then assumed higher values of *d*_*E*_ and repeated the above exercise until we found parameters where viremia became undetectable on the same timescale but before the first reported time point past the EOT for the two patients. We examined how model predictions of viral load changes during treatment compared with patient data to ensure consistency with the best‐fit parameters identified above. Note that parameters defining CTL exhaustion during HCV infection remain unknown. Our goal was not to estimate the latter parameters precisely, but instead to examine whether the immunologic hypothesis was consistent with data of viral load changes in patients experiencing EOT^+^/SVR.

### Bistability and the estimation of the minimum required treatment duration

We found roots of the nonlinear algebraic equations corresponding to the steady states of the model equations when ε = 0 for a range of values of the parameters quantifying CTL effector function and exhaustion. Root finding was done using Mathematica. In particular, we explored the influence of *m* and constructed a bifurcation diagram to illustrate the steady states of the system. We performed linear stability analysis to assess the stability of the steady states. We identified parameter regimes where two stable states, chronic infection and viral clearance, were separated by an unstable intermediate steady state. Treatment that allowed crossing the unstable boundary from the chronic infection state and ensured that viremia stayed across the boundary following the cessation of treatment would yield EOT^+^/SVR. Next, using parameter values spanning the above regimes, we solved model equations (Equations (1), (2), (3), (4)) with ε > 0 for different treatment durations *τ*_*d*_. For each parameter combination, we identified the minimum *τ*_*d*_ required to yield EOT^+^/SVR within 3000 days of the start of treatment. SVR was defined as achieved when the viral load dropped below the cure boundary of 1 virion in 15 L.

## Conflict of Interest

The authors declare that they have no conflicts of interest.
