# Supplementary material for: Modeling how reversal of immune exhaustion elicits cure of chronic hepatitis C after the end of treatment with direct‐acting antiviral agents
Source: Immunol Cell Biol. 2018 Jun 5;96(9):969–80. doi: 10.1111/imcb.12161 (PMC6220890; doi:10.1111/imcb.12161)
Supplement: Supplementary file 6 [file IMCB-96-969-s006.docx]

**Supplementary Information**

**Modelling how reversal of immune exhaustion elicits cure of chronic hepatitis C after the end of treatment with direct-acting antiviral agents**

**Subhasish Baral^1^, Rahul Roy^1,2,3^, Narendra M. Dixit^1,2,#^**

^1^Department of Chemical Engineering, Indian Institute of Science, Bangalore

^2^Centre for Biosystems Science and Engineering, Indian Institute of Science, Bangalore

^3^Molecular Biophysics Unit, Indian Institute of Science, Bangalore

^#^**Correspondence:**

Narendra M. Dixit

Department of Chemical Engineering, Indian Institute of Science, Bangalore, Karnataka, India 560012

Telephone: +91-80-2293-2768; Fax: +91-80-2360-8121

Email: narendra@iisc.ac.in; nmdixit@gmail.com

**SUPPLEMENTARY TEXT NOTES**

**Supplementary text 1. Mathematical model with target and infected cell proliferation**

To account for cell proliferation, we replaced Equations 1 and 2 of our model (see text) with the following equations^1-3^:

$\frac{dT}{dt}=s+r_{T}T\left(1-\frac{T+I+N}{T_{m}}\right)-d_{T}T-\beta VT$ (S1.1)

$\frac{dI}{dt}=\beta VT+r_{I}I\left(1-\frac{T+I+N}{T_{m}}\right)-\omega I-mEI$ (S1.2)

where *r_T_* and *r_I_* are the proliferation rates of target and infected cells, respectively, *N* are cells that cannot be infected due, for instance, to inadequate entry receptor expression^4^ or other factors involved in viral replication, and *T_m_* is the carrying capacity. The other equations (Equations 3 and 4) remained unaltered.

The model (Equations S1.1 and S1.2 and Equations 3 and 4), using realistic parameter estimates (Supplementary figure 1), described patient data in Figure 3a well. We applied this model to the data in Figure 3a because the data displays a low second phase slope, which in previous studies^1-3^ has been attributed to cell proliferation. In the model without cell proliferation, the low second phase slope is attributed to weak effector killing of infected cells (low *m*). The model with proliferation allowed a low second phase slope despite strong effector killing by allowing replenishment of infected cells via proliferation. Accordingly, after the EOT, the model predicted rapid viral load reduction, leading to SVR within weeks, which appears more realistic^5, 6^ than the best-fit without proliferation, which predicted high viremia last over extended durations past the EOT before SVR could be achieved (Figure 3a). We computed the steady states and found that the model with cell proliferation also exhibited bistability (Supplementary figure 1). More frequent measurements past the EOT would help distinguish between these models.

**Supplementary text 2. Mathematical model with cumulative antigenic stimulation for exhaustion**

Following previous formalisms^7, 8^, we replaced Equation 4 of our model (see text) with the following equations to allow for cumulative antigenic stimulation leading to CTL exhaustion:

$\frac{dE}{dt}=\lambda+b_{E}\frac{I}{k_{B}+I}E-\xi\frac{Q^{n}}{q_{c}^{n}+Q^{n}}E-\mu E$ (S2.1)

(S2.2)

where *Q* is a measure of cumulative stimulation, *d_q_* its normalized decay rate, *φ* the half-maximal antigen level for cumulative stimulation, *ξ* the exhaustion rate, *q_c_* the half-maximal cumulative stimulation for exhaustion, and *n* the Hill coefficient for exhaustion. The other equations (Equations 1-3) remained unaltered. Here, we set *d_q_=*1/day, and *φ=*10^4^ cells mL^-1^.

We fit the model (Equations 1-3, S2.1, and S2.2) to patient data, following the same method as in Figure 3, and found that the model provided good fits to the data (Supplementary figure 2a). We computed the steady states of the system and found that this model also exhibited bistability (Supplementary figure 2b).

**Supplementary text 3. Linear stability analysis**

Defining the vector $\overrightarrow{x}=\left(T,I,V,E\right)$ of dependent variables in our model, we derived the Jacobian matrix, $J\left(\overrightarrow{x}\right)$, with elements $\left\{J\right\}_{ij}=\partial\left(dx_{i}/dt\right)/\partial x_{j}$. We then computed the eigenvalues of $J\left(\overrightarrow{x^{*}}\right)$ for each of the steady states $\overrightarrow{x^{*}}$ and examined their real parts. The real parts of all the eigenvalues had to be negative for linear stability. The real parts of the eigenvalues are presented in Supplementary figure 3.

**SUPPLEMENTARY REFERENCES**

1. Dahari H, Ribeiro RM, Perelson AS. Triphasic decline of hepatitis C virus RNA during antiviral therapy. Hepatology 2007;**46**:16-21.

2. Padmanabhan P, Garaigorta U, Dixit NM. Emergent properties of the interferon-signalling network may underlie the success of hepatitis C treatment. Nat Commun 2014;**5**:3872.

3. Rong L, Dahari H, Ribeiro RM, Perelson AS. Rapid emergence of protease inhibitor resistance in hepatitis C virus. Sci Transl Med 2010;**2**:30ra2.

4. Padmanabhan P, Dixit NM. Mathematical model of viral kinetics in vitro estimates the number of E2-CD81 complexes necessary for hepatitis C virus entry. PLoS Comput Biol 2011;**7**:e1002307.

5. Malespin M, Benyashvili T, Uprichard SL,et al. Prevalence of end of treatment RNA-positive/sustained viral response in HCV patients treated with sofosbuvir combination therapies. Therap Adv Gastroenterol 2017;**10**:68-73.

6. Sarrazin C, Wedemeyer H, Cloherty G, et al. Importance of very early HCV RNA kinetics for prediction of treatment outcome of highly effective all oral direct acting antiviral combination therapy. J Virol Methods 2015;**214**:29-32.

7. Conway JM, Perelson AS. Post-treatment control of HIV infection. Proc Natl Acad Sci U S A 2015;**112**:5467-5472.

8. Johnson PLF, Kochin BF, McAfee MS, et al. Vaccination alters the balance between protective immunity, exhaustion, escape, and death in chronic infections. J Virol 2011;**85**:5565-70.

**SUPPLEMENTARY FIGURE LEGENDS**

**Supplementary figure 1. Predictions of the model including cell proliferation.** Bifurcation diagram indicating stable (black) and unstable (grey) steady state viral loads as functions of the strength of the effector response, *m*, using the model in Supplementary text 1. The parameter values employed are similar to previous studies^1-3^: *s*=7.3 cells/mL/day, *β*=8×10^-7^ mL/cells/day, *r_T_*=*r_I_*=1/day, *T_m_*=10^7^ cells/mL, *N*=0.8*T_m_*, *d_E_*=8/day, *k_D_*=6.5×10^4^ cells/mL*.* The other parameters are the same as in Table 1. With these parameters and with *m*=1.53 mL/cells/day, the model described the data in Figure 3a.

**Supplementary figure 2. Predictions of the model with cumulative antigenic stimulation for exhaustion. (a)** Model predictions (solid lines) (see Supplementary text 2) compared with the same patient data as in Figure 3a (symbols). The best-fit parameter estimates and their 95% CIs are as follows: *δ*=0.059 (0.026-0.091) /day, *ε*=0.9991 (0.9988-0.9994), and *c*=6.3 (5.8-6.8) /day. These yielded *p*=4.4 virions/cell/day and *m*=0.31 mL/cells/day. The minimal values of *d_E_*=8/day and *q_c_*=0.7780 were employed. **(b)** Bifurcation diagram indicating stable (black) and unstable (grey) steady state viral loads as functions of the strength of the effector response, *m*, with the same model and other parameters as in **(a)**.

**Supplementary figure 3. Sensitivity analysis.** Partial rank correlation coefficients (PRCCs) indicating the sensitivity of the sum of square errors (SSE) between our model predictions of viral load and patient data from Figure 3a to variations in model parameter values. The asterisks indicate PRCCs significantly different from the dummy (*P* < 0.01). For these calculations, we adapted the MATLAB codes available on Dr. Denise Kirschner’s website (<http://malthus.micro.med.umich.edu/lab/usadata>). The model was simulated for 500 days before treatment initiation to rule out parameter combinations yielding spontaneous clearance. We fixed the production rate of virions by infected cells, *p*, based on the initial viral load (*p=cV*_0_/*I*_0_). *V*_0_ and *I*_0_ are the initial steady state viral load and infected cell population, respectively. The remaining parameters were varied 2-10 fold for calculating PRCCs.

**Supplementary figure 4. Linear stability of steady states.** Real parts of the eigenvalues of the Jacobian matrix corresponding to the steady states in Figure 5a (see Supplementary text 3). Each subplot corresponds to a different steady state: high viral load stable **(a)**, intermediate viral load unstable **(b)**, low viral load unstable **(c)** and low viral load stable **(d)**. The different line types correspond to the different eigenvalues.

**Supplementary figure 5. Bistability with alternative parameter values.** Bifurcation diagrams akin to those in Figure 5a, but with *n*=1 and **(a)** *k_B_*=10^3^ cells/mL and **(b)** *k_B_*=10^4^ cells/mL. Other parameters remain the same as in Figure 5a.
